# The Effect of Energy Densities on the Shear Bond Strength of Self-Adhering Flowable Composite to Er:YAG Pretreated Dentin

**DOI:** 10.1155/2016/6507924

**Published:** 2016-10-17

**Authors:** Paul Nahas, Toni Zeinoun, Zeina Majzoub, Karim Corbani, Samir Nammour

**Affiliations:** ^1^Department of Restorative and Esthetic Dentistry, Lebanese University, Campus Rafic Hariri, Hadath, Beirut, Lebanon; ^2^Department of Oral and Maxillo-Facial Surgery, Faculty of Dentistry, Lebanese University, Campus Rafic Hariri, Hadath, Beirut, Lebanon; ^3^Department of Periodontology, Lebanese University, Campus Rafic Hariri, Hadath, Beirut, Lebanon; ^4^Department of Operative Dentistry, School of Dentistry, Saint Joseph University, Beirut, Lebanon; ^5^Department of Dental Sciences, Faculty of Medicine, University of Liege, Liege, Belgium

## Abstract

*Objective*. To investigate the shear bond strength of self-adhering flowable resin composite, to dentin, after exposing it to Er:YAG laser radiation, at different energy densities.* Materials and Methods*. Sixty freshly extracted human third molars were randomly divided into five groups (*n* = 12). In the control group, dentin was left unirradiated, whereas, in the other four groups, dentin was irradiated with Er:YAG laser in noncontact mode (MSP mode = 100 *µ*s; 10 Hz; beam diameter: 1.3 mm; speed of 1 mm/second; air 6 mL/min; and water 4 mL/min), and respectively, with the following level of energy (50 mJ, 60 mJ, 80 mJ, and 100 mJ). Then, self-adhering flowable resin composite was bonded to all prepared dentin surfaces. Shear bond strength (SBS) was applied and fractured surfaces were examined using scanning electron microscopy.* Results*. SBS values showed significant differences in 60 mJ (*P* < 0.05) compared to other groups. Morphological evaluation revealed tags or plugs in dentinal tubules, especially when 60 mJ and 80 mJ were used. All four groups tended to leave more residues on the dentin surface, than the control group.* Conclusion*. Er:YAG dentin irradiation may enhance SBS of the self-adhering flowable resin composite when it is used at the appropriate low level of energy density.

## 1. Introduction

During the last decade, adhesive systems improvement—ranging from three steps to a single step—had a direct impact on practitioner's daily clinical applications. Although three-step adhesive systems were known to be the gold standard for adhesion to enamel and dentin [1], new adhesives tended to replace conventional method for their ease of use and clinical timesaving procedure. Other than self-etch bonding systems, few self-adhesive composites were introduced to the market; the performance of their bond strength was unsatisfactory when compared to gold standard or self-etch bonding systems [2, 3]. Mahdan et al. have demonstrated that pulpal pressure and smear layer characteristics negatively affected the initial bond strengths of one-step self-etch adhesives to dentin [4–8]. Moreover, two-step self-etch and one-step self-etch bonding systems and self-adhesive composite (SAC) require phosphoric acid etching prior to bonding in order to achieve clinically acceptable SBS values especially on enamel [3, 8–10]. Therefore, removing the smear layer by whatever means is intrinsic when attempting to improve bond strength results [3, 5, 11].

The most effective dental lasers on hard tissue are the erbium lasers. Lasers, such as erbium-doped yttrium aluminium garnet (Er:YAG) and erbium, chromium-doped yttrium, scandium, gallium, and garnet (Er,Cr:YSGG), were introduced earlier than 1997 but first dental practices had begun in 1997 according to literature [12]. When erbium lasers were used in fast cutting dental hard tissues, a high level of energy was applied. This caused fusion of dentin surface. Thus, it compromised the restoration bond strength [13–16]. Subsequently, based on the work of Ekworapoj et al. and Bahrami et al., preparing dentin surface for bonded restoration requires low-level laser energy to help eliminate smear layer and open dentin tubules [12, 13, 17]. Dentin irradiation, using erbium laser with temperature reaching values around 300°C, partially denatures collagen structure [18, 19], whereas when using fluency below 1.94 J/cm^2^ effects, such as water loss, organic matrix (collagen) degradation, and OH^−^ increase, appear to be more intense in the central irradiated area and on the superficial tissue layer. Affected area is less intense in the surrounded irradiated surface and deeper in the tissue layers [18]. This thermal damage increases gradually with the intensification of laser energy [20] and when temperature exceeds 225°C. Collagen fibers start to denature and no reversion to initial conformation is observed after rehydration [19].

It was demonstrated that Ca/P ratio of an irradiated Er:YAG laser was significantly lower than the theoretical value for hydroxyapatite. This indicates a decline in the concentration of Ca. This phenomenon is similar to decalcification of the tooth [20]. Moreover, Brulat et al. mentioned that dentin conditioning by low fluence with Er:YAG laser improved adhesion of composite resin to dentin [17, 21]. Yet, results showed that the absence of smear layer formation during dentin preparation by Er:YAG laser did not improve the adhesion values of self-etching adhesive systems [22, 23]. Nevertheless, Yazici et al. have proven that dentin prepared with Er:YAG laser might improve bonding effectiveness to dentin [10, 19, 24, 25], similar to using phosphoric acid prior to applying SACs [25–27]. However, some other studies have reported opposite results; studies showed that laser irradiation weakens the bond strength of the adhesive [28] and that conventional etching with 35% phosphoric acid yielded significantly higher bond strength values compared to thermal etching with the Er:YAG laser [29].

Also, irradiation distance could affect shear bond strength. So, increasing the distance would decrease the negative effects of laser irradiation [30]. It was demonstrated in previous studies that high level of energies increased dentin microhardness in the deepest area of the cavity until 60 *μ*m [31]. Consequently, the shear bond strength, in surfaces with lower energy density, was high due to fewer morphologic changes [32]. Bonding self-adhering flowable resin composite to laser irradiated dentin still remains a challenge because of the lack of information about alterations in collagen fibrils and mineral content promoted by Er:YAG laser irradiation.

The results of shear bond strength tests of composite bonded to Er:YAG pretreated dentin are still controversial. One of the possible causes may be the variability of irradiation conditions in published studies and the difficulty in standardizing the pressure on applied composites to dentin during curing and bonding protocol.

The purpose of our preliminary in vitro study was to determine whether different low level of Er:YAG laser energy densities when preparing dentin helps improve values of shear bond strength of self-adhering flowable composite. The null hypothesis tested was that there is no difference in the shear bond strength of self-adhering resin composite to dentin when four levels of laser density energy were used.

## 2. Materials and Methods

Our study was conducted in accordance with the recommendations of the ethics committee of our university. Sixty freshly extracted human third molars were selected free from any restoration, fracture, cavities, or whatever pathology that could affect the expected bond strength results. Immediately after the extraction, teeth were stored for one week in a 0.1% thymol solution at room temperature for disinfection. They were then washed abundantly with filtered water, hand-scaled, and embedded into fast setting autopolymerizing resin acrylic (Paladur, Heraeus Kulzer, Inc., South Bend, IN, USA) with the crown extending 2 mm above the enamel-cementum junction. Flat dentin surfaces were obtained after a transversal cut of the crowns at a distance of 4 mm from the occlusal plane (IsoMet 2000, Buehler®, Ltd., IL, USA) using a slowly rotating diamond blade (250 rpm) with 100 grams of load. The smear layer was standardized by wet-grounding dentin flat surfaces, respectively, with 320 grit and then 600 grit silicon carbide paper (Matador, Germany). All samples were checked under a stereomicroscope (Leica/Meyer Instruments, Houston, USA) confirming the absence of exposed pulp or remaining enamel except at the periphery.

**Figure 1:**
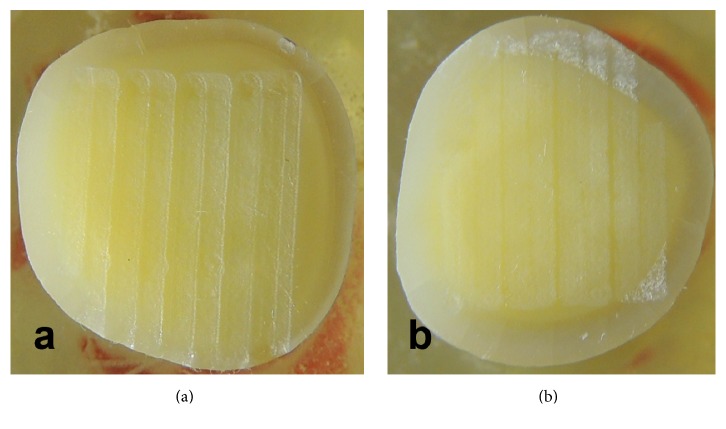
Impact of laser on dentin using a computer driven robot.

The 60 teeth were randomly divided into 5 groups. Dentin of the first group (control, *n* = 12) did not receive any laser irradiation. The other 4 groups (*n* = 12) were irradiated with Er:YAG laser wavelength of 2.940 nm (Fidelis; Fotona, Medical Laser, Ljubljana, Slovenia) in a noncontact mode using handpiece H14 with Micro-Short Pulse mode (MSP, pulse duration: 100 *μ*s), frequency of 10 Hz, under air/water spray (air, 6 mL/min, and water, 4 mL/min), and 1.3 mm as a beam diameter at the impact point, with, respectively, the energy levels and fluency of 50 mJ (17.692 J/cm^2^), 60 mJ (20.769 J/cm^2^), 80 mJ (25.385 J/cm^2^), and 100 mJ (31.769 J/cm^2^) (Table 1) and with an irradiation speed of 1 mm/second. This was done using a custom made 2D motion robot driven by stepper motors connected to a computer through Universal Serial Bus port. Figures 1(a) and 1(b) show the automated impact and the uniformly irradiated dentin of different laser energy levels, excluding possible variation of irradiation between samples.

**Figure 2:**
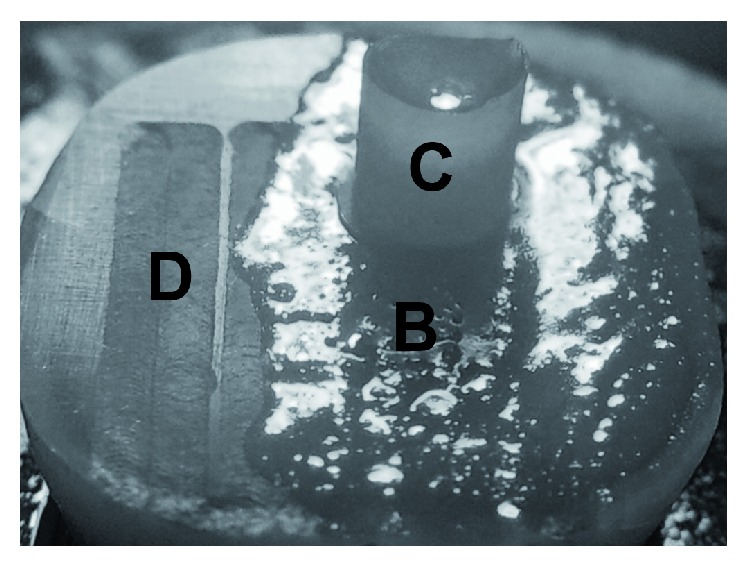
Almost half of the irradiated dentin received self-adhering flowable resin composite with the 2.38 × 2 mm cylindrical build-up. The rest of the dentin was left unbounded for SEM observation. D: dentin irradiated without self-adhering flowable resin composite over irradiated dentin; C: cylindrical build-up.

According to the manufacturer's instructions, all samples received a thin layer of self-adhering flowable resin composite (Vertise Flow, Kerr, Orange, CA, USA) by rubbing half of the irradiated dentin surface for 15 to 20 seconds with the proprietary microbrush and then light cured for 20 seconds (Demi Plus LED Light Curing System, Kerr, USA). The other half of the irradiated dentin surface was left without any bonding treatment for scanning electron microscope (SEM) observation. A cylinder with a diameter of 2.38 mm and a height of 2 mm of self-adhering flowable resin composite was added over the first light-cured layer using a special bonding mold insert (Ultradent Products, Inc., South Jordan, UT, USA) (Figure 2). All samples were stored in distilled water at room temperature for 24 hours before performing the shear bond strength test.

Shear bond strength test was applied on all samples using a notched edge in a testing machine (Ultradent Products, Inc., South Jordan, UT, USA) at a crosshead speed of 1.0 mm/min. Results were obtained in megapascal from the peak load at failure. Dentin surfaces were examined with a stereomicroscope (Leica/Meyer Instruments, Houston, USA) at 20x magnification to determine the failure mode.

The modified adhesive remnant index (ARI) proposed by Ostby et al. [23] was employed to assess the amount of adhesive left on the dentin surfaces as follows:

 Score 1: all of the adhesives remained on the tooth
 Score 2: more than 90% of the adhesives remained on the tooth
 Score 3: 10–90% of the adhesives remained on the tooth
 Score 4: less than 10% of the adhesives remained on the tooth
 Score 5: no adhesive remained on the tooth

### 2.1. Specimen Preparation for SEM Observation

Each sample served for double SEM observation. Part of the irradiated dentin that did not receive any self-adhering flowable resin composite treatment served as control showing the impact of laser on sound dentin. The second observation was performed to the zone that received the SBS tests. All samples were dehydrated in a graded series of aqueous ethanol (25%, 50%, 75%, 90%, and 100%), respectively, for 20 seconds for the first three groups and then for 30 seconds for the fourth group and for one hour for the last ethanol concentration (100%). Samples were left 24 hours for their final dehydration. Dentin surfaces were gold-sputtered and observed for topographical changes under SEM at 500x to 2500x magnification. The data were finally analyzed using one-way ANOVA and Tukey's test (*P* = 0.05) using SPSS software ver. 21.0 (SPSS, Co., Ltd., Tokyo, Japan).

## 3. Results

### 3.1. Descriptive Results

**Figure 3:**
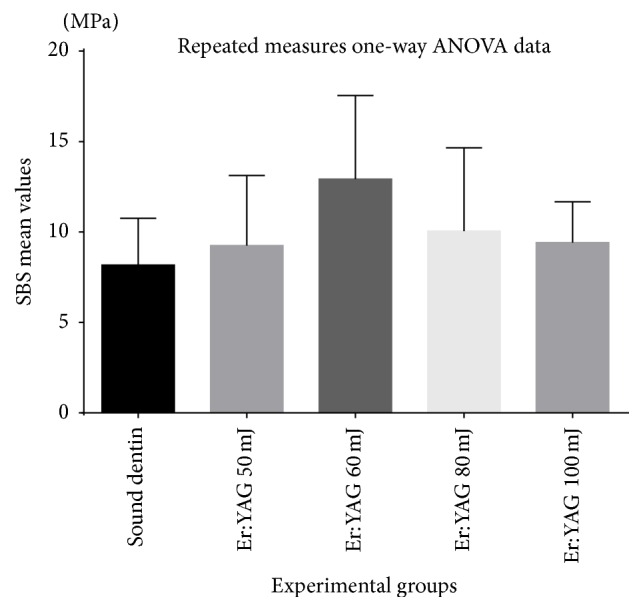
Bar graph showing the mean values with the standard deviations.

The means and the standard deviations for SBS are exhibited in Table 1. Group 3 representing 60 mJ of energy exhibited the highest mean with 12.9167 MPa; then again values decrease at a higher level of energy (Figure 3). When examining failure mode in fractured specimens as they are represented in Table 2, score 5 and score 4—where no adhesive or only 10% of adhesives remain on dentin—appear to be the most common failure mode. Stereomicroscopic observation resulted in adhesive dominance in comparison to cohesive failure.

### 3.2. Analytical Results

Bond strength data were submitted to statistical analysis. One-way ANOVA and Tukey's test showed a statistically significant difference between control group and irradiated dentin when using Er:YAG at 60 mJ of energy (*P* < 0.05) (Table 3).

### 3.3. SEM Topographical Observation

**Figure 4:**
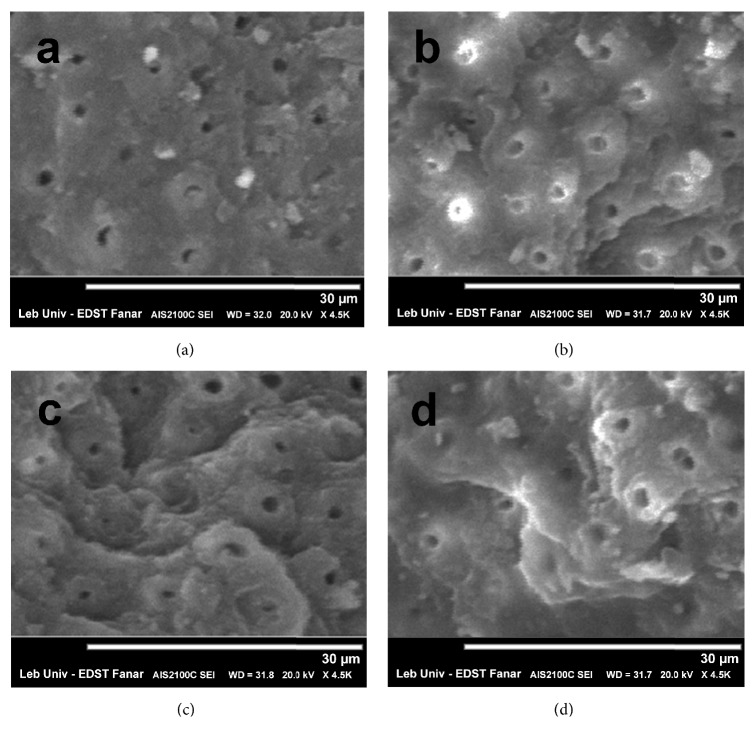
SEM observation after irradiation with Er:YAG and before bonding self-adhering flowable resin composite, respectively, with (a) 50 mJ, (b) 60 mJ, (c) 80 mJ, and (d) 100 mJ.

SEM examination of dentin surface after laser irradiation showed what is presented in Figure 4.

The smear layer appears to be clean at all levels. Tubules are open, especially for 60 mJ and 80 mJ (Figures 4(b) and 4(c)). There is no presence of any cracks or melted dentin in all figures. When using 60 mJ (Figure 4(b)) peritubular dentin is less affected, while laser impact with 80 mJ and 100 mJ (Figures 4(c) and 4(d)) seems to be more effective on inter- and peritubular dentin.

**Figure 5:**
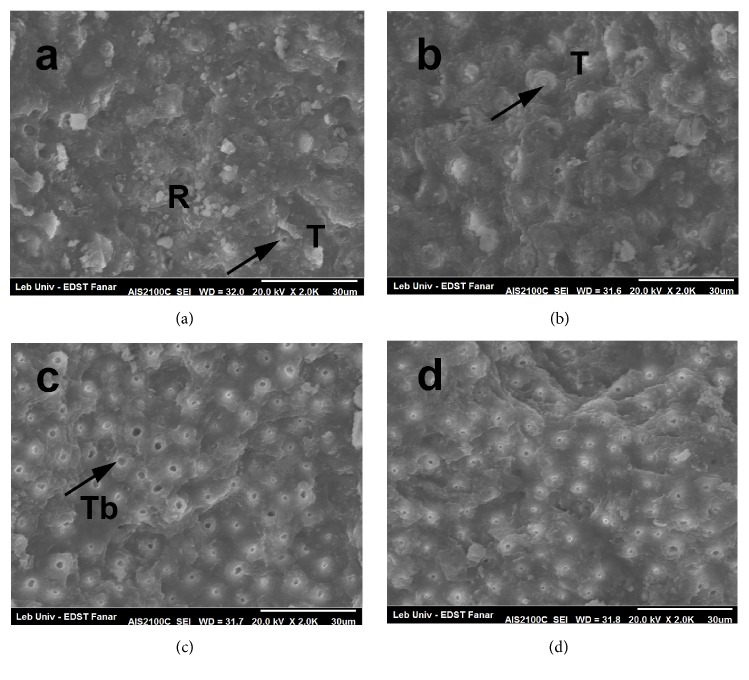
SEM observation after SBS done on dentin surfaces bonded with self-adhering flowable resin composite. (a) shows cohesive fracture with resin composite remnants; some tubules are open without any visible tags or plugs inside. (b) shows adhesive fracture; dentin tubules are visible. Most of them are filled with resin tags or plugs; fracture occurred between tags and composite; peritubular collagen fibrils are visible. (c) shows typical adhesive fracture; dentin is totally visible; tubules are open with less resin composite tags or plugs inside, signifying less infiltration of self-adhering flowable resin composite inside dentin tubules; and peritubular collagen fibrils are visible. (d) shows a large amount of dentin tubules with smaller opening and typical adhesive fracture; and fewer tags or plugs infiltrated dentin tubules. R: resin composite; T: tags or plugs; Tb: dentin tubules.

SEM examination of dentin surface after SBS tests showed the following:

1. At very low level of energy (50 mJ), the resin composite is not totally eliminated, and a small number of tubules appear (Figure 5(a)).
2. At a higher level of energy (60 mJ), the smear layer is eliminated, and a large number of tubules are visible, with most of them filled with resin tags (Figure 5(b)).
3. When a larger amount of energy is used (80 mJ), the smear layer is completely removed; a large number of tubules are visible, with some of them containing resin tags, while others are empty with a small diameter (Figure 5(c)).
4. When a higher level of energy is used (100 mJ), the smear layer is completely removed; the dentin tubules opening seems to be very small and part of the tubules is free of resin composite (Figure 5(d)).

## 4. Discussion

In this study, standardized flat surfaces of sound dentin were employed for testing self-adhering flowable resin composite bond strength when prepared with Er:YAG at different levels of energies. Flowable composite was used to avoid any difference in the pressure of composite paste during the curing procedure. Unprepared dentin surfaces were considered as the control group. The acidic phosphate group of the chosen self-etch flowable resin composite etches the tooth structure and creates chemical bonds with the calcium. However, the acidity of the product was insufficient to eliminate the smear layer. This smear layer prevents the diffusion of resin into the dentin structure (collagen layer), hence affecting SBS values [29]. Consequently, it is advisable to eliminate the smear layer in order to improve the infiltration of the flowable composite into superficial collagen fibers and dentin tubules.

Previous studies have proven that dentin treatment with laser at a low level of energy is effective in eliminating the smear layer. It may allow a reduction in the microfissures and roughness of the irradiated surfaces [33]. Some studies mentioned that when using high energy laser tends to melt the irradiated dentin, alters and denatures collagen fibers, closes tubules, and prevents bonding infiltration in opened tubules and the formation of resin tags [12]. Tuloglu et al. [34] evaluated the shear bond strength of conventional and self-adhering resin composite to dentin and they found that SBS values of self-adhering resin composite are lower than conventional ones whether they are bonding to lacteal or permanent teeth. Moreover Russo et al. [35] concluded that Er:YAG laser irradiation prior to bonding has increased the SBS values of all adhesive systems used including self-adhering resin composite. That was evident in our study when SEM observation combined with SBS values showed net amelioration at low level of energy (60 mJ) compared to values coming from a higher one. Our results correlate with Bahrami et al.'s study [13], where tensile bond strength values at low fluency were improved even though a different type of bonding system was used; it demonstrated that laser at low energy of 80 mJ helps eliminate denatured collagen fibrils, melted, fused, and weakly attached to adjacent dentin. Such dentin represents porous layer of melted minerals, which forms microfissures that could partially be infiltrated by adhesive [24].

Results from the failure mode correlate with SBS values and SEM observation regarding the enhancement of self-adhering flowable resin composite bond quality. Meanwhile, almost all dentin surfaces for sound or unirradiated dentin scored a total absence of composite when observed with a stereomicroscope using 20x magnifications [2]. The increase in remnants left when laser irradiation was performed would explain the effect of demineralization of laser, while the decrease of remnants on unirradiated surfaces explains the reduced etching effect of self-adhering flowable resin composite on dentin. This would correlate with the studies of Shahabi et al.; they concluded that if the remnant is increased or cohesive fracture occurred, SBS values should increase, explaining the amount of dentin demineralization effect of the technique [36]. These findings prove that the etching effect of self-adhering flowable resin composite is relatively weak and should, therefore, be enhanced; they also prove that laser cavity preparation is seemingly a promising technic. Yazici et al. [19] compared two different types of dentin preparation: SiC paper and Er:YAG irradiation. They concluded that laser treatment increased dentin-bonding values of self-adhering flowable resin [9].

Stereomicroscopic observation showed deeper morphological alteration of dentin. However, SBS values did not increase, respectively, by increasing energy levels. When the same dentin was examined under SEM, the smear layer was visibly washed away and tubules were opened similar to a low level of energy. However, one exception was observed: peritubular dentin is less affected by the laser yielded to less opened tubules, preventing resin composite from infiltrating into dentin structure (Figure 1). Bachmann et al. have reported that when heating human and bovine dentin temperature increased to a certain point above 225°C which would denature collagen fibers, irreversibly yielding to weak bonding with resin composite [14, 15]. Other studies have reported that microhardness of dentin decreases subsequent to laser irradiation when using high level of energy due to the increase in temperature and alteration of the physical and chemical composition [12].

One ought not to compare our results to others due to the number of variables used in the study: bonding agents, laser parameters starting from energy level to frequency mode, and the amount of spray (water/air) that could modify the laser thermal effect on dentin.

In conclusion, the null hypothesis is only rejected when using Er:YAG at 60 mJ. It is important to understand that using high level of energy for excavating caries and cutting enamel and dentin does not necessarily prepare tooth hard tissues for bonding procedure. We tried to prove in our study the importance of laser low level of energy based on a combination of power, frequency, water, and air which can dissolve the smear layer without destroying the remaining collagen fibers by enhancing the shear bond strength of self-adhering flowable resin composite to dentin.

Additional studies might be of interest in order to study the stability of the obtained bond strength within time.

## 5. Conclusion

Within the limitation of this study, we concluded that Er:YAG laser beam used for dentin preparation can increase the quality of shear bond strength of self-adhering flowable resin composite when used at the appropriately delivered energy density (60 mJ, 10 Hz in MSP, and noncontact mode of 1.3 mm tip diameter) under air/water cooling.

## Figures and Tables

**Table 1:** Mean shear bond strength (in megapascals) and standard deviation for the experimental groups.

Experimental group	Energy selected	Frequency (Hz)	Energy density (J/cm^2^)	Mean (MPa) ± SD
Group 1 (control)	No irradiation	—	—	08.1667 ± 2.59837
Group 2	Er:YAG 50 mJ	10	17.692	09.2417 ± 3.87895
Group 3	Er:YAG 60 mJ	10	20.769	12.9167 ± 4.62441
Group 4	Er:YAG 80 mJ	10	25.385	10.0500 ± 4.60583
Group 5	Er:YAG 100 mJ	10	31.769	09.4167 ± 2.25301

**Table 2:** Predominant failure patterns (in percent) by stereomicroscope under ×20 magnification (*n* = 12/group).

Experimental group	Failure mode				
	Score 1 (%)	Score 2 (%)	Score 3 (%)	Score 4 (%)	Score 5 (%)
Group 1 (control)	0 (0%)	0 (0%)	0 (0%)	2 (16.68%)	10 (83.32%)
Group 2	0 (0%)	1 (8.34%)	2 (16.68%)	5 (41.64%)	4 (33.34%)
Group 3	0 (0%)	0 (0%)	1 (8.34%)	7 (58.33%)	4 (33.33%)
Group 4	0 (0%)	0 (0%)	0 (0%)	4 (33.33%)	8 (66.67%)
Group 5	0 (0%)	0 (0%)	1 (8.34%)	8 (66.66%)	3 (25%)

**Table 3:** Statistical comparison for the experimental groups.

Experimental group	Mean difference	Std. error	Sig.
No laser treatment versus Er:YAG 50 mJ	−1.07500	1.48008	0.998
No laser treatment versus Er:YAG 60 mJ	−4.75000^*∗*^	1.48008	0.045^*∗*^
No laser treatment versus Er:YAG 80 mJ	−1.88333	1.48008	0.937
No laser treatment versus Er:YAG 100 mJ	−1.25000	1.48008	0.995

^*∗*^The mean difference is significant at the 0.05 level.
